# Neuroprotective effects of coenzyme Q10 on neurological diseases: a review article

**DOI:** 10.3389/fnins.2023.1188839

**Published:** 2023-06-23

**Authors:** Shokufeh Bagheri, Rasool Haddadi, Sahar Saki, Masoumeh Kourosh-Arami, Masome Rashno, Ali Mojaver, Alireza Komaki

**Affiliations:** ^1^Department of Neuroscience, School of Science and Advanced Technologies in Medicine, Hamadan University of Medical Sciences, Hamadan, Iran; ^2^Department of Pharmacology, School of Pharmacy, Hamadan University of Medical Science, Hamadan, Iran; ^3^Department of Neuroscience, School of Advanced Technologies in Medicine, Iran University of Medical Sciences, Tehran, Iran; ^4^Asadabad School of Medical Sciences, Asadabad, Iran; ^5^Student Research Committee, Asadabad School of Medical Sciences, Asadabad, Iran

**Keywords:** Alzheimer's disease, depression, epilepsy, Parkinson's disease, neurological disorder

## Abstract

Neurological disorders affect the nervous system. Biochemical, structural, or electrical abnormalities in the spinal cord, brain, or other nerves lead to different symptoms, including muscle weakness, paralysis, poor coordination, seizures, loss of sensation, and pain. There are many recognized neurological diseases, like epilepsy, Alzheimer's disease (AD), Parkinson's disease (PD), multiple sclerosis (MS), stroke, autosomal recessive cerebellar ataxia 2 (ARCA2), Leber's hereditary optic neuropathy (LHON), and spinocerebellar ataxia autosomal recessive 9 (SCAR9). Different agents, such as coenzyme Q10 (CoQ10), exert neuroprotective effects against neuronal damage. Online databases, such as Scopus, Google Scholar, Web of Science, and PubMed/MEDLINE were systematically searched until December 2020 using keywords, including review, neurological disorders, and CoQ10. CoQ10 is endogenously produced in the body and also can be found in supplements or foods. CoQ10 has antioxidant and anti-inflammatory effects and plays a role in energy production and mitochondria stabilization, which are mechanisms, by which CoQ10 exerts its neuroprotective effects. Thus, in this review, we discussed the association between CoQ10 and neurological diseases, including AD, depression, MS, epilepsy, PD, LHON, ARCA2, SCAR9, and stroke. In addition, new therapeutic targets were introduced for the next drug discoveries.

## Introduction

Neurological diseases endanger human health and lifestyle. Neurological diseases affect a large number of people all over the world (World Health Organization, 2006). Stroke accounts for the death of over six million people annually, and more than 80% occur in low- and middle-income countries (Akinyemi et al., 2021). Over 50 million individuals in the world suffer from epilepsy (Scott et al., 2001). Also, there are 47.5 million cases of dementia, including 7.7 million new patients each year (Trandafir, 2016). Alzheimer's disease (AD) is the commonest cause of dementia accounting for 60–70% of dementia cases (Huang et al., 2020). Migraine affects over 10% of the world's population (Steiner et al., 2014).

**Figure 1:**
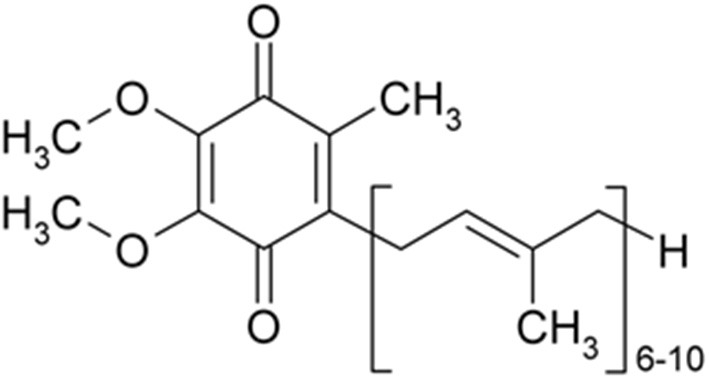
CoQ10 is a 1,4-benzoquinone and Q represents the quinone chemical group. Its tail contains 10 isoprenyl chemical subunits (image from the PubChem database).

Several causes and mechanisms have been suggested for neurological disorders (Urdinguio et al., 2009). Lifestyle, genetics, infections, diet, environmental factors, and physical damage have been revealed as the causes of neurological disorders (World Health Organization, 2006). Neurological disorders are associated with the following physical symptoms: partial or full paralysis, seizures, muscle weakness, partial or full loss of sensation, reading and writing disabilities, poor cognitive functions, unexplainable pain, and reduced alertness (Stone and Carson, 2011). Accordingly, oxidative stress and imperfective energy metabolism can be regarded as the pathogenesis of many neurodegenerative diseases, such as Parkinson's disease (PD), AD, multiple sclerosis (MS), epilepsy, depression, and stroke (Choonara et al., 2009). The prevalence of age-dependent disorders has recently been increasing (Bigal et al., 2006). Coenzyme Q10 (CoQ10) is a strong neuroprotective agent in neurodegenerative disorders. The mechanisms of CoQ10 on neurological disorders are shown in Table 1. The levels of CoQ10 diminish in the brain and different tissues in animals and humans with age; thus, CoQ10 has an effective therapeutic role in age-related neurodegenerative disorders (Spindler et al., 2009; Kadian et al., 2022). CoQ10 is also known as ubiquinone, ubidecarenone, CoQ10, CoQ, or Q10. It is a 1,4-benzoquinone and Q represents the quinone chemical group (Figure 1). In this review, the neuroprotective effects of CoQ10 on neurological diseases, including AD, depression, epilepsy, MS, PD, stroke, autosomal recessive cerebellar ataxia 2 (ARCA2), Leber's hereditary optic neuropathy (LHON), and spinocerebellar ataxia autosomal recessive 9 (SCAR9) were discussed (Table 2).

**Table 1:** The effect of CoQ10 on neurological disorders.

**Function of CoQ10**	**Neurological disorder**
↓Apoptotic death	AD
↓TBARS	AD
↑Antioxidant enzymes	AD
↓MDA, LPO	AD
↓Inflammation	AD
↑Cholinergic functioning	AD
↑TAC, SOD, GPx	BD
↓LPO, MDA	BD
↓NF-kB, p38, JNK	BD
↑5-HT_1A_, 5-HT_2A_ receptors, (p)GSK-3β, CREB, pCREB, BDNF	BD
↓IL-1β, IL-2, IL-6, TNF-α	BD
↓Kainate neurotoxicity	Epilepsy
↓Apoptosis	Epilepsy
↓iNOS and eNOS expression	Epilepsy
↓Endothelial NO generations	Epilepsy
↓TNF-α, IL-10	MS
↑GPx, SOD, TAC	MS
↓MDA	MS
↓LPO damage	PD
↓α-synuclein accumulation	PD
↓TNFα and proinflammatory cytokines	Stroke
↓Stress oxidative	Stroke

↑, Increasing.

↓, Decreasing.

**Table 2:** The doses of CoQ10 and intervention duration.

**References**	**Test models**	**Treatment arms**	**Disease**
Ishrat et al. (2006)	Animal models (male Wistar rats)	CoQ10 (10 mg/kg b.w., i.p.) for 3 weeks	Alzheimer's disease
Yang et al. (2009)	Animal models (male Lewis rats)	2% creatine + 1% CoQ10 for 1 week	Alzheimer's disease, Parkinson's disease –Huntington
Komaki et al. (2019)	Animal models (male Wistar rats)	CoQ10 (50 mg/kg, i.p.) for 3 weeks	Alzheimer's disease
Forester et al. (2015)	Human models (adult)	CoQ10 (500 mg/day) for 2 weeks	Bipolar disorder
Morris et al. (2013)	Human models (adult)	CoQ10 (25, 50, 100, and 150 mg/kg/day) for 3 weeks	Depression
Sanoobar et al. (2016)	Human models (women and men)	CoQ10 (500 mg/day) for 12 weeks	Depression-multiple sclerosis
Erol et al. (2010)	Animal models (male Wistar rats)	CoQ10 (10 mg/kg, i.p) for 30 min	Ischemia/reperfusion injury
Oztay et al. (2007)	Animal models (male mice)	CoQ10 (1.5 mg/kg) daily for 15 days	Hyperthyroidism
Guo et al. (2011)	Animal models (rat)	CoQ10 (25 mg/kg) for 12 weeks	Vascular endothelial abnormalities
Sattarinezhad et al. (2014)	Animal models (male albino mice)	CoQ10 (50, 100, 200, and 400 mg/kg, p.o.) for 7 days	Tonic seizures
Lee D.-H. et al. (2012)	Human models (adult)	CoQ10 (60 and 150 mg) for 12 weeks	Coronary artery diseases (CAD)
Ibrahim Fouad (2020)	Animal models (adult rat)	CoQ10 (10 mg/kg b.w)	Multiple sclerosis
Somayajulu-Nitu et al. (2009)	Animal models (adult rat)	CoQ10 (50 mg/kg b.w) for 7 weeks	Parkinson's disease
Muthukumaran et al. (2014)	Animal models (C57BL/6 mice)	Ubisol- Q10 (50g/ml and 150 g of PTS/ml) for six days	Parkinson's disease
Cleren et al. (2008)	Animal models (mice)	CoQ10 (1,600 mg/kg) for 3 months	Parkinson's disease
Attia and Maklad (2018)	Animal models (mice)	CoQ10 (200 mg/kg) for 3 weeks	Parkinson's disease
Belousova et al. (2016)	Animal models (rat)	CoQ10 (30 mg/kg) for 60 min	Cerebral ischemia
Obolenskaia et al. (2020)	Animal models (rat)	CoQ10 (30 mg/kg) for 45 min	Cerebral ischemia
Nasoohi et al. (2019)	Animal models (rat)	CoQ10 (200 mg/kg) for 24 h	Stroke
Kuo et al. (2001)	Human models (adult) (adult)	CoQ10 (90, 160, and 200 mg) for 4 months	Leber's hereditary optic neuropathy (LHON)
Chariot et al. (1999)	Human models (men)	CoQ10 (250 mg) tocopherol (500 mg), vitamin K3 (10 mg), vitamin C (150 mg), thiamine (10 mg), and riboflavin (10 mg) over 1 year	LHON
Shalata et al. (2019)	Human models (adult)	CoQ10 (500 mg twice a day) for 3 weeks	Spinocerebellar ataxia autosomal recessive 9 (SCAR9) and Autosomal Recessive Cerebellar Ataxia 2 (ARCA2)
Weyer et al. (1997)	Human models (adult)	CoQ10 (30 or 90 mg) for 6 months	Alzheimer's disease
Gutzmann and Hadler (1998)	Human models (women and men)	CoQ10 (90 mg) for another 12 months	Alzheimer's disease

### Biosynthesis of CoQ10 and its neuroprotective/antioxidant effect on neurotoxicity

CoQ should reduce itself following oxidization, which is done using various NAD(P)H oxidoreductases in the plasma membrane, like NAD(P)H: quinone oxidoreductase 1, NADH-cytochrome b5 reductase, or NADPH-CoQ reductase (Rashid et al., 2021). Accordingly, CoQ should be distributed among them, which is regulated by particular proteins (Hidalgo-Gutiérrez et al., 2021; Kemmerer et al., 2021). CoQ is a lipid-soluble compound in the inner mitochondrial membrane (IMM). IMM separates the mitochondrial matrix from the intermembrane space and is an environment for electron transport in the respiratory chain (Sharaf, 2017). In IMM, CoQ can accept electrons from FADH2 through succinate dehydrogenase complex II (CII) and/or NADH through NADH ubiquinone oxidoreductase complex I (CI). The electrons are transported to cytochrome c via CoQH2-cytochrome c reductase complex III (CIII), and cytochrome c can transfer the electrons to the oxygen via cytochrome c oxidase complex IV (CIV). Electron transportation among these complexes is associated with the pumping protons toward the intermembrane space, producing a proton motive force used by the ATP synthase complex V (CV) to generate ATP (Lodish et al., 2008). Electron transportation in the mitochondrial complexes of the respiratory chain (CI, CII, CIII, CIV, and CV) is done by the generation of super-complexes, a supramolecular organization joining the individual complexes in the mitochondria in a structure, where CoQ is an important component (Sharaf, 2017). The CoQH2/CoQ ratio as a sensor for the mitochondria metabolic status modulates electron flow direction in the mitochondrial respiration as well as the generation of mitochondrial complexes/super-complexes (Guaras et al., 2016). CoQH2/CoQ ratio plays a key role in using the alternative oxidase (AOX) to accept electrons from CoQ and cause a reduction in the CoQH2/CoQ ratio, leading to a decrease in rearranged during transfection (RET) and ROS generation (Szibor et al., 2020).

Mitochondria as dynamic organelles alter their shape, size, number, and location in response to environmental changes, in the health state. In the disease state, fission and fusion, as mitochondrial dynamics, exhibit some alterations. In mitochondrial fission, the mitochondria face division and two mitochondria are fused into one for mutual advantage (Chan, 2012). The absence of fission results in mitochondrial dysfunction, including mitochondria interconnection and elongation and motility loss toward the cell periphery. The absence of fusion causes mitochondrial fragmentation as well as ultrastructural impairments and consequently, dysfunction (Srivastava, 2017). Alterations in mitochondrial dynamics are controlled by some proteins. Dynamin-related protein 1 (Drp1) and fission protein 1 (Fis1) are two important fission protein markers. Drp1 is the main regulator of mitochondrial fission and Fis1 is a partner protein of Drp1 (Losón et al., 2013).

Orally administrated water-soluble CoQ10 enhanced bioavailability compared to lipid-soluble CoQ10 (Cui et al., 2021). Water-soluble CoQ10 is not natural and can be prepared artificially. The natural CoQ10 is lipid-soluble (Wear et al., 2021). CoQ can inhibit mitochondrial fission and improve mitochondrial dynamics by decreasing Drp1 and Fis1 proteins (Li et al., 2017). Moreover, treatment with CoQ10 inhibits mitochondrial fission in hydrogen peroxide-treated astrocytes of the optic nerve head (ONH) (Noh et al., 2013). Furthermore, CoQ10 prevents the trauma-induced phosphorylation of Drp1 and blocks the fission-induced activity of Drp1 (Zhang et al., 2021). CoQ10 partially inhibits the astrocyte mitochondrial structure against oxidative stress-related mitochondrial fission (Moreira et al., 2010). Moreover, Some CYP450 isoforms, such as CYP 2D6 or 2E1, may be involved in the development of neurodegenerative diseases. In an *in vitro* model, CYP induction causes neurorepair. The toxic effect of MPP+ on cell viability in undifferentiated neuroblastoma SH-SY5Y cells treated with the CYP inducers, β-naphthoflavone (βNF) and ethanol (EtOH), before and during exposure to the parkinsonian neurotoxin, was rescued by both βNF and EtOH treatments. Neuroprotective effect of CYP inducers was due to a decrease in ROS production, restoration of mitochondrial fusion kinetics, and mitochondrial membrane potential (Fernandez-Abascal et al., 2020). Furthermore, the hydroxy analog of CoQ10 can be produced by cytochrome P450 (CYP450) of mitochondria (Slowik, 2019).

Ultraviolet B irradiation causes the augmentation of ROS, which is highly toxic to many types of cells and leads to lipid peroxidation (LPO), protein oxidation, and mutagenesis (Pathak et al., 2019). ROS-induced damage can be prevented by CoQ10 in the neuronal cells and astrocytes. Therefore, CoQ10 stabilizes the mitochondrial membrane potential, protects the mitochondria from oxidative damage, improves mitochondrial respiration, inhibits the mitochondria-mediated cell death pathway, and activates mitochondrial biogenesis (Jing et al., 2015). Furthermore, CoQ10 by scavenging ROS protects neurons against oxidative stress in several neurodegenerative disorders and protects ONH structures and astrocyte components (Nakazawa et al., 2019). Prokaryotes and eukaryotes have similar CoQ10 biosynthesis: a long polyisoprenoid lipid tail attaches to a benzenoid precursor, followed by modifying the benzenoid ring through successive steps to obtain the ultimate product (Pierrel et al., 2022). In eukaryotes and some prokaryotes, the isoprene carbon units are obtained from the mevalonate pathway to make the CoQ side chain (Fernández-del-Río and Clarke, 2021), or the deoxyglucose-5-phosphate pathway in plants, prokaryotes, and some protozoa (Wang and Hekimi, 2016). CoQ using a long polyisoprenoid tail is anchored at the phospholipid bilayer midplane. Mutations in several *CoQ* and *PDSS* genes are linked to primary CoQ_10_ deficiency, while mitochondrial DNA (mtDNA) mutations result in secondary CoQ_10_ deficiency. CoQ5 and CoQ9 proteins are found in many mitochondrial protein complexes in human 143B cells and *CoQ9* and *CoQ5* knockdown inhibits CoQ_10_ levels (Yen et al., 2020). There are some antibodies and mitochondrial localizations of mature proteins for such proteins, except CoQ2 and PDSS1. There are also some PDSS2 and CoQ3 isoforms. PDSS1, CoQ3, and PDSS2 are involved in preserving the stability of the other proteins (Chen et al., 2013). In the mitochondria, some protein complexes contain CoQ3, CoQ4, CoQ6, CoQ7, or PDSS2 protein. There are two specific PDSS2-containing protein complexes. Their transient knockdown, except *CoQ8* and *CoQ6*, reduced CoQ_10_ levels, but just *CoQ7* knockdown could hamper mitochondrial respiration and elevated ubiquinol to ubiquinone ratios and also cause the accumulation of a putative biosynthetic intermediate characterized by reversible redox property, like CoQ_10_ (Yen et al., 2016). Also, PDSS2 suppressed the concentrations of different CoQ proteins (not CoQ3 and CoQ8A) that can be detected in cybrids consisting of the pathogenic mtDNA A8344G mutation or in 143B cells treated with carbonyl cyanide p-trifluoro-methoxyphenyl hydrazone (FCCP), which is consistent with our previous results for CoQ5 (Yen et al., 2011, 2016). These new findings may shed light on the possible centome of CoQ synthome in human cells as well as the understanding the role of PDSS and CoQ proteins in pathological and physiological conditions (Wang et al., 2022). The CoQ_10_ and CoQ levels showed a negative correlation with malignancy degree and a positive correlation with citrate synthase (CS) activity, while PDSS2 levels showed a positive correlation with malignancy. Also, lower mitochondrial DNA-encoded cytochrome c oxidase subunit 2 levels showed no association with a higher malignancy degree and lower CoQ protein levels. Mitochondrial abnormalities are linked to defected CoQ_10_ maintenance in the progression of human astrocytoma (Yen et al., 2020). Homozygous mutations in humans in both genes caused severe neuromuscular disease, with nephrotic syndrome observed in *PDSS2* deficiency. Presumed autoimmune kidney disease with the missense *Pdss2*^*kd*/*kd*^ genotype is possibly because of a mitochondrial CoQ biosynthetic defect in mice (Yen et al., 2022).

CoQ10 deficiencies are genetically and clinically heterogeneous. The syndrome has five main clinical phenotypes: (1) cerebellar ataxia, (2) severe infantile multisystemic disease, (3) encephalomyopathy, (4) isolated myopathy, and (5) nephrotic syndrome. In some cases, pathogenic mutations are observed in genes associated with the CoQ10 biosynthesis (primary CoQ10 deficiencies) or those not directly associated with CoQ10 biosynthesis (secondary CoQ10 deficiencies). The pathogenesis of primary CoQ10 deficiencies has been linked to respiratory chain defects, ROS generation, and apoptosis variably (Peng et al., 2008). Primary deficiency due to mutations in genes is associated with CoQ10 biosynthesis. Secondary deficiency is possibly associated with hydroxymethylglutaryl coenzyme A (HMG-CoA) reductase inhibitors (statins), which are used to treat hypercholesterolemia. CoQ10 dietary contributions are very small; however, supplementation can increase plasma CoQ10 levels (Quinzii and Hirano, 2011). CoQ10 is highly safe with limited adverse events. Several clinical trials have been done using some CoQ10 doses. Adverse gastrointestinal effects, such as nausea, are not due to the active ingredient because of no reported dose-response relationship. Daily intakes of 1,200 mg do not cause adverse effects than the dose of 60 mg (Potgieter et al., 2013). According to the “observed safe level” risk assessment method, CoQ10 is safe at up to 1,200 mg/day (Hathcock and Shao, 2006). CoQ10 plays a role in the reduced International Normalized Ratio (INR) in patients who use warfarin (Shalansky et al., 2007). Engelsen et al. (2003) reported alterations in prothrombin time, and INR levels are not important in patients on stable, long-term warfarin therapy receiving 100 mg of CoQ10 for 4 weeks. Mitochondrial cells have oxidized (ubiquinone) and reduced (ubiquinol) species of CoQ10. Ubiquinol is an antioxidant and is oxidized to ubiquinone in free radical reactions, limiting LPO. The ubiquinol reverse reduction activates endogenous regeneration systems (tocopherol and ascorbate) (Gille et al., 2008). The ratio between oxidized and reduced CoQ10 species is a biomarker for oxidative stress *in vivo* (Kalenikova et al., 2018). The ubiquinone therapeutic effectiveness has been reported in many diseases affecting this pathogenetic factor (Chan et al., 2015). Ubiquinol is the reduced form of CoQ10, associated with antioxidant function. Hence, the tissues and cells should have molecular mechanisms to recover their active form, including the dihydroorotate dehydrogenase action in the IMM, causing pyrimidine biosynthesis and reducing ubiquinone through the oxidation of dihydroorotate to rotate. In addition, the CoQ10 involvement in the flavoprotein/electron transfer of flavoprotein to ubiquinone ratio is needed in the oxidoreductase system, making the ubiquinol able to be recovered by involvement in the oxidation of the fatty acids (Littarru and Tiano, 2010). The stepwise one-electron oxidation of the two hydroxyl groups on the benzoquinone ring leads to three redox states of CoQ: the fully reduced ubiquinol (UQH2), the half-reduced ubisemiquinone radical (UQH• in the protonated form), and the fully oxidized ubiquinone (UQ) (Cedeno, 2010). Also, its tail contains ten isoprenyl chemical subunits (Matthews et al., 1998; Geromel et al., 2002). CoQ10 is a crucial cofactor to produce ATP in the electron transport chain (ETC) (Manzar et al., 2020). This coenzyme delivers electrons from complexes I and II and transfers them to complex III (Alcázar-Fabra et al., 2016). Moreover, an increase in the expression of mitochondrial uncoupling proteins (UCPs) demonstrates the antioxidant role of CoQ10 (Persson et al., 2012). CoQ_10_ as an important endogenous antioxidant is a crucial component of the mitochondrial respiratory chain (MRC). CoQ molecules are dynamically divided in a pool attached to and engulfed by the super-complexes I + III, and another pool related to complex II or other mitochondrial enzymes using CoQ as a cofactor. Such a CoQ-free pool can be applied by enzymes linking the MRC to other pathways, like the fatty acid β-oxidation and amino acid catabolism, pyrimidine *de novo* biosynthesis, proline, arginine, and glyoxylate metabolism, glycine metabolism, and sulfide oxidation metabolism that some of them are attached to metabolic pathways in other compartments (Pradhan et al., 2021). The antioxidant function of CoQ10 is because of its completely reduced ubiquinol form. Thus, a CoQ10 deficiency can cause some diseases due to a failure in energy metabolism and compromising cellular antioxidant capacity. Several diseases are caused by CoQ10 deficiency, such as heterogeneous MRC disorders. Defects in cellular CoQ10 status are due to its primary or secondary deficiency (Neergheen et al., 2019; Hargreaves, 2021).

Based on these findings, the antioxidant impact of the CoQ10 declines the function of inflammatory factors evidenced by gene expression analysis as well as cell culture assessments (Hargreaves, 2021). The robust neuroprotective effects of the CoQ10 on neurotoxicity have been shown in many *in vitro* investigations and also animal models of neurological diseases (Spindler et al., 2009).

## Methods

Online databases, such as Scopus, Google Scholar, Web of Science, and PubMed/MEDLINE were systematically searched until December 2020 using keywords, such as review, neurological disorders, and CoQ10.

### Effect of CoQ10 on AD

In 2017, an estimated 700,000 American people aged ≥ 65 years were found with AD when they died (Alzheimer's Association, 2017). AD is also known as the prevalent type of dementia (Wang et al., 2014). Deficits in memory and learning, defects in thinking, and behavioral signs are the symptoms of dementia (Alzheimer's Association, 2013; Shekarian et al., 2020). Recently, genetic (Picone et al., 2016a) and environmental (Chen et al., 2009; Picone et al., 2016b) risk factors were found effective in the pathogenesis of AD. The accumulation of Aβ senile plaques plays an important role in AD pathogenesis (Golde et al., 2000; Kowalska, 2004). It Presenilin 1 (PS-1) mutation (L235P: proline by leucine substitution at codon 235) causes the formation of Aβ42 and Aβ40 in the brains of transgenic mice and cultured cells (Borchelt et al., 1996; Duff et al., 1996) and leads to AD (Schellenberg et al., 2000; Xia, 2000; Yang et al., 2008). CoQ10 diminished plaque pathology in an amyloid precursor protein (APP)/PS1 mouse model of AD. Therefore, CoQ10 might be a therapeutic candidate for the treatment of AD (Yang et al., 2010).

According to *in vitro* investigations, Aβ induces oxidative stress. For instance, Aβ increases the hydrogen peroxide (H2O2) and lipid peroxide concentrations in cultured cells, and antioxidants, like vitamin E with a protective role in neurons against Aβ-related cytotoxicity (Chan and Shea, 2006). Oxidative stress can cause aging and results from the imbalance between oxidants and antioxidants (Wang et al., 2014; Jiang et al., 2016; Butterfield, 2018; Shekarian et al., 2020). Accordingly, oxidative stress causes AD (Markesbery, 1997; Santos et al., 2004; Swerdlow, 2012; Bonda et al., 2014). Both mitochondrial dysfunction and oxidative damage lead to Aβ deposition in AD (Beal, 2004). CoQ10 is capable of scavenging free radicals (Gazdík et al., 2003; Gvozdjáková et al., 2022). The synthesis of CoQ10 is diminished in older people (Borek, 2004). CoQ10 inhibited apoptotic death and damage caused by ROS (Li et al., 2014) and improved AD (Dumont et al., 2011). Oral administration of CoQ10 (10 mg/kg b.wt., i.p. in corn oil) in AD rats reduced thiobarbituric acid reactive substances (TBARS) and elevated the activity of antioxidant enzymes in the brain (Ishrat et al., 2006). Vinpocetine (VIN) and CoQ10 (200 mg/kg, suspended in saline) in combination with physical and mental activities caused a significant attenuation in the neurodegeneration due to AlCl3 administration by improving AD markers in brain tissue and inflammatory and oxidant markers (Ali et al., 2019). Treatment of hypercholesterolemia rats with Co-Q10 alone or in combination with omega-3 (1,000 mg) regulated cholinergic functioning, reduced brain inflammation and oxidative stress, and increased the functional outcome verified through the histopathological evaluation of brain tissues (Ibrahim Fouad, 2020).

**Figure 2:**
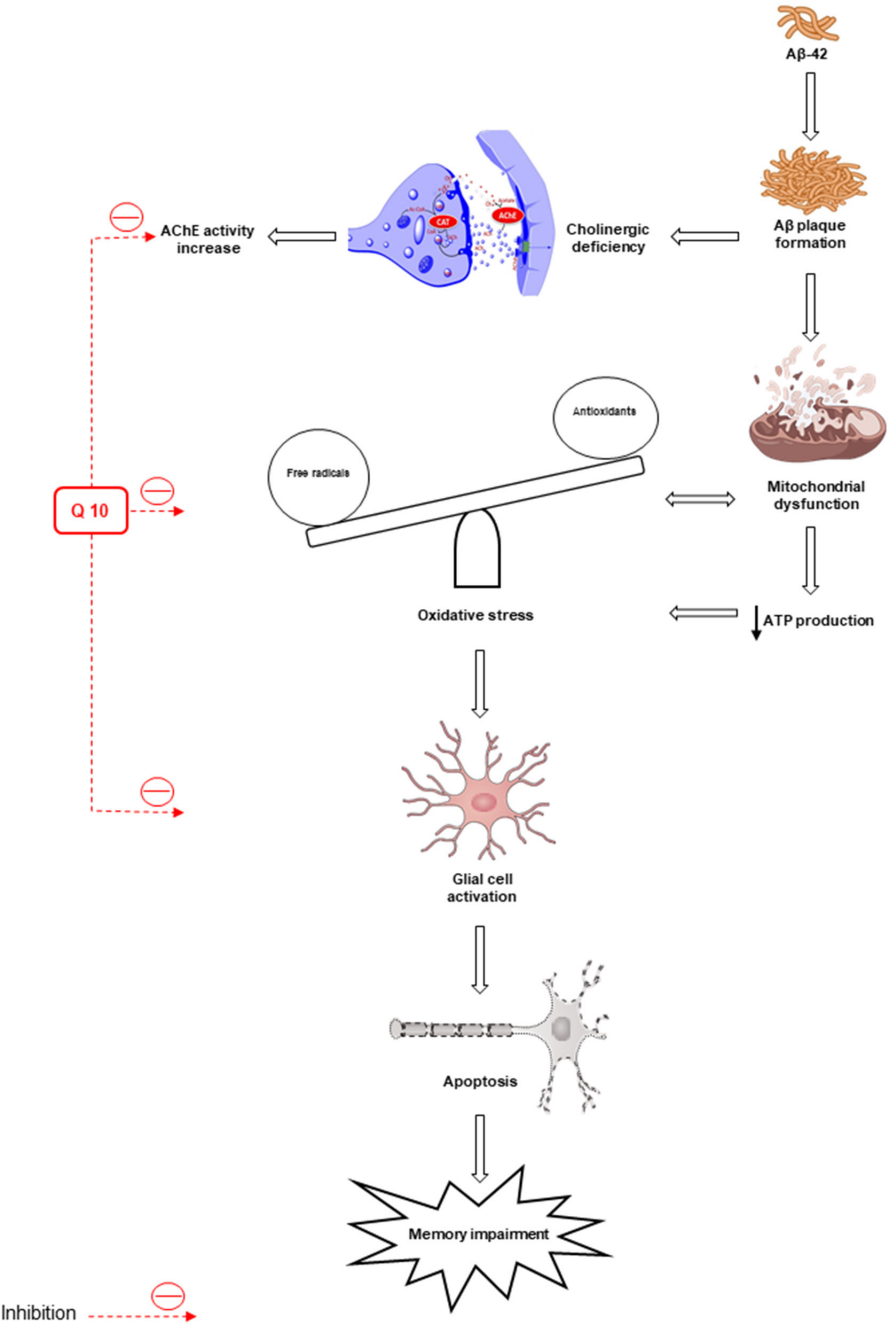
Oxidative stress and mitochondrial dysfunction lead to the formation of β-amyloid (Aβ) senile plaques. Deposition of Aβ increases the activity of acetylcholinesterase (AChE). Also, oxidative stress leads to apoptosis and memory impairment by activating glial cells. The antioxidant and anti-apoptotic effects of CoQ10 improve memory. AIF, apoptosis-inducing factor; AchE, acetylcholinesterase.

The synaptic mechanisms of learning and memory in vertebrates are studied by long-term potentiation (LTP) of the hippocampal synaptic transmission (Bliss and Collingridge, 1993). The long-term synaptic plasticity in the hippocampus (HIP) is suppressed by Aβ peptides (Chen et al., 2000; Asadbegi et al., 2016; Ramezani et al., 2020a). Ubisol-Q10 in drinking water (at ~6 mg/kg/day) could decrease circulating Aβ peptide, improve long-term memory, preserve working spatial memory, and inhibit Aβ plaque generation in transgenic mice aged 18 months compared to untreated transgenic mice (Muthukumaran et al., 2018). The effects of oral administration of CoQ10 (50 mg/kg/oral gavage/daily) on the hippocampal synaptic plasticity in animals subjected to Aβ injection were examined by field potential recording methods and the results showed an improvement in memory and neuroplasticity of neurons following CoQ10 administration (Komaki et al., 2019) (Figure 2).

The administration of CoQ10 alone or in combination with other antioxidants improved learning and memory and prevented oxidative stress, inflammation, and cellular death in various models of AD and frontotemporal dementia, including aged rodents with aluminum-induced AD, rats with forebrain lesions, rats receiving ICV infusion of Aβ-42 or STZ, transgenic mice with different mutations inducing AD or frontotemporal dementia, and cell cultures using different human or rodent cells (Jiménez-Jiménez et al., 2023). Initial short-term randomized clinical trials have shown an improvement in several neuropsychological tests in AD patients treated with CoQ10 in comparison with those receiving the placebo due to the potential effectiveness of CoQ10 (Weyer et al., 1997; Gutzmann and Hadler, 1998). A systematic review was conducted to examine the possible therapeutic effects of CoQ10 in experimental models of AD and other dementias, as well as in humans with AD and mild cognitive impairment. The potential role of CoQ10 treatment in AD and improving memory in aged rodents in experimental models deserves future studies on patients with AD through the assessment of other causes of dementia and mild cognitive impairment (Jiménez-Jiménez et al., 2023).

### Effect of CoQ10 on depression

Depression as a neurological disorder is caused by the lack of serotonin (Anderson and Maes, 2014). Lower serotonin levels can be a major outcome of tryptophan metabolism shift to kynurenine formation (Caspi et al., 2003; Oxenkrug, 2010). The kynurenine pathway degrades more than 95% of tryptophan (a precursor to serotonin). In tryptophan oxidation, indoleamine 2, 3-dioxygenase 1 (IDO 1) is a rate-limiting enzyme. The tryptophan-to-kynurenine conversion plays a role in the development of depression (Dantzer et al., 2008). Most people experience repeated episodes of mood disorders, psychosocial morbidity, and high use of healthcare services, which persist into later life (Bartels et al., 2000). Bipolar depression is the significant and least effectively treated stage of bipolar disorder (BD). BD is an aging-associated disease and longer periods of BD are spent depressed instead of having manic/hypomanic and cycling/mixed symptoms (Kalin, 1996; Judd et al., 2002, 2003). In BD, the proportion of the time spent in depressive episodes is more than the time spent in manic episodes (Forester et al., 2015). Treatment of BD has not been widely considered and also treating depressive symptoms is not easily achievable in the manic stage (Konradi et al., 2004).

Lower plasma CoQ10 concentrations in depressed patients were compared with healthy controls (Maes et al., 2009; Lesser et al., 2013). Treatment with CoQ10 (400 mg/d titrated up by 400 mg/d every 2 weeks up to 1,200 mg/d) diminished depression severity in patients with BD (Forester et al., 2012). Also, CoQ10 is effective in the pathophysiology of many disorders linked to depressive symptoms, like fibromyalgia (FM), major depression, and myalgic encephalomyelitis (Forester et al., 2012; Aboul-Fotouh, 2013). CoQ10 [25, 50, 100, and 150 mg/kg/day, i.p dissolved in 1% dimethyl sulfoxide (DMSO)] was injected into the HIP for 40 days, and its effect was assessed on serotonin levels in platelets of cases with FM. The results showed improved depressive symptoms compared to cases treated with the placebo (Alcocer-Gómez et al., 2014). CoQ10 (50, 100, and 200 mg/kg/day, dissolved in 1 % DMSO) received for 6 weeks exhibited significant antidepressant effects indicated by a significant decrease in stress-induced alterations in the forced swim test (FST) and open field test, and also a decrease in corticosterone levels and the weight of adrenal glands (Abuelezz et al., 2017). A high dose of CoQ10 (500 mg/day) caused an improvement in depression in older adults with BD (Forester et al., 2012).

**Figure 3:**
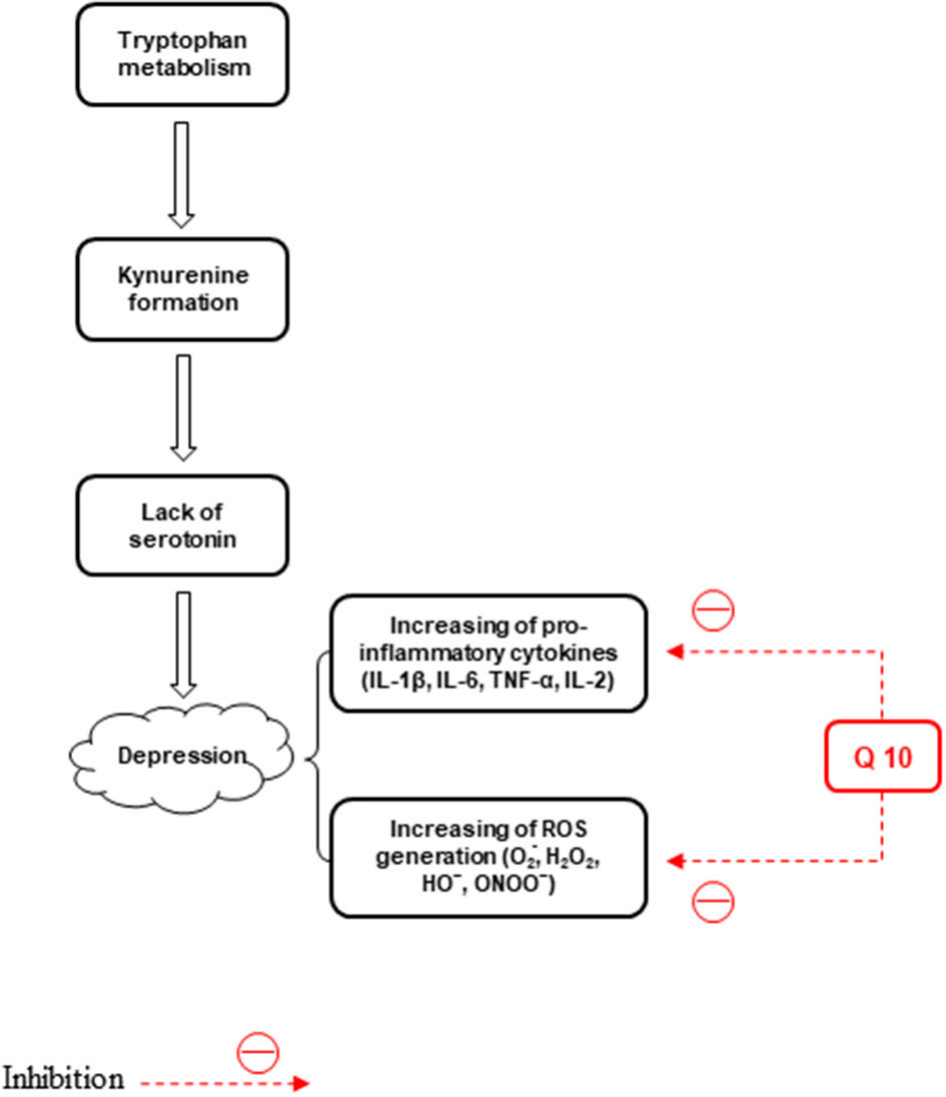
The effects of CoQ10 on depression by inhibiting inflammation and stress oxidative pathways.

Mitochondrial dysfunction, oxidative stress, and inflammation are involved in the BD pathophysiology. Inflammatory responses, such as elevated leucocyte and neutrophil counts and plasma concentrations of proinflammatory cytokines and their receptors are found in cases of severe depression. On the other hand, major depression is linked to reduced antioxidant levels as well as induced nitrosative and oxidative pathways (Maes et al., 2011). ROS, like hydroxyl radical, superoxide, H2O2, and peroxynitrite, are effective in the pathogenesis of major depression (Lucca et al., 2009a,b) (Figure 3). It CoQ10 plays a role in depression pathophysiology through the anti-inflammatory and neuroprotective properties and suppresses the generation of pro-inflammatory cytokines (Schmelzer et al., 2008; Mohamed and Said, 2021). Furthermore, the anti-inflammatory, antioxidant, and mitochondrial modulatory effects of CoQ10 (200 mg/d) administrated for 8 weeks are involved in its effectiveness in the treatment of BD patients (Mehrpooya et al., 2018). Reduced levels of CoQ10 are associated with an elevated level of tumor necrosis factor-alpha (TNF-α) and oxidants, such as ROS (Schmelzer et al., 2008; Leonard and Maes, 2012). In another study, treatment with CoQ_10_ (500 mg/kg/day, gavage) reduced LPO and elevated GSH levels and total antioxidant capacity (TAC) values, and SOD and glutathione peroxidase (GPx) activities in both brain areas of mice. The suppressed neuro-inflammatory response in the prefrontal cortex (PFC) and HIP was observed, evidenced by reduced NF-kB, p38, and JNK concentrations in the CoQ_10_ groups (Salehpour et al., 2019). Moreover, the CoQ10 antioxidant effect was exhibited by its capability to significantly decrease elevated hippocampal 4-hydroxynonenal and MDA levels and increase the decreased catalase and glutathione levels. Furthermore, CoQ10 caused a significant reduction in the levels of different pro-inflammatory cytokines, such as interleukin-1β (IL-1β), IL-2, IL-6, and TNF-α (Abuelezz et al., 2017). Therefore, CoQ10 due to its anti-inflammatory and anti-oxidative effects can be used to treat depression.

Effect of CoQ10 (100 mg/kg/day) or/and fluoxetine (10 mg/kg/day) was assessed on mRNA expression, 5-HT_1A_ and 5-HT_2A_ receptors, GSK-3β, phosphorylated (p)GSK-3β, CREB, pCREB, and BDNF protein expression in rats receiving chronic unpredictable mild stress (CUMS) for 6 weeks (Abuelezz et al., 2018). Recently, we assessed the effects of CoQ_10_ (10 mg/kg, dissolved in corn oil) on behavioral dysfunction and CoQ_10_ levels in the rat brain. A significant difference was found between the depression induced by streptozotocin (STZ) and control groups tested by the splash test and FST 24 h after STZ treatment. In addition, according to the validated and accurate high-performance liquid chromatography (HPLC), reduced CoQ_10_ levels were found in the brain of the STZ group (Andalib et al., 2019). Supplementation with CoQ10 (500 mg/day) could improve depression in bipolar patients (Forester et al., 2012).

### The effects of CoQ10 on epilepsy and seizures

Epilepsy is the commonest neurological disease worldwide, in which spontaneous unusual electrical discharges of neurons are observed throughout the brain (Patel, 2002). Temporal lobe epilepsy (TLE) is the commonest form of epilepsy in adults that is commonly linked to hippocampal sclerosis, neurodegeneration, and hippocampal circuit reorganization (Jokeit and Schacher, 2004). An animal model of TLE was designed by unilateral intrahippocampal kainic acid injection in rodents. It was a “post-status” model where epilepsy can spread following the agent-induced status epilepticus (SE) (Löscher, 2002). These models are used to study the effects of possible antiepileptic agents because in the time interval between the status and the first spontaneous seizures, we can examine the effect of neuroprotective and prophylactic agents on epilepsy (Sharma et al., 2007). Epilepsy approximately affects 0.5–1% of the general population (Hauser et al., 1991). Contrary to the prevalence of recent effective antiepileptic agents in cases with epilepsy, novel antiepileptic agents are used with stronger anticonvulsant activity (Sattarinezhad et al., 2014). Therefore, new therapeutic strategies have been established to prevent or even reverse the molecular and cellular mechanisms of epileptogenesis (Löscher and Schmidt, 2006).

CoQ10 caused a reduction in the kainate-related model of epilepsy (Yalcin et al., 2004). CoQ10 has anti-apoptotic and antioxidant effects (Papucci et al., 2003). Moreover, CoQ10 (0.01, 0.1, and 1 mM) received for 3 weeks exerted a neuroprotective effect on the HIP against kainate neurotoxicity *in vitro* (Won et al., 2011; Kumar et al., 2022), and blunts cell death and cellular apoptosis in the hippocampal CA3 area after SE (Chuang et al., 2009). The neuroprotective properties of CoQ10 (i.p. at 10 mg/kg/day) dissolved in normal saline in the intrahippocampal kainate model of TLE have not yet been identified and are under investigation (Baluchnejadmojarad and Roghani, 2013).

Nitric oxide (NO) is a regulator of seizure activity because of its various anticonvulsant (Starr and Starr, 1993; Theard et al., 1995; Tsuda et al., 1997) and proconvulsant (Osonoe et al., 1994; Van Leeuwen et al., 1995; Nidhi et al., 1999) effects depending on the seizure type, the source of NO, and other neurotransmitter system contentions. Treatment with CoQ10 (10 mg/kg i.p) decreased inducible and endothelial NO generations in a rat with testicular ischemia/reperfusion injury (Erol et al., 2010). Also, CoQ10 (1.5 mg/kg, dissolved in serum physiologic administrated daily for 15 days by gavage) showed a decreasing effect on increasing induced- and endothelial nitric oxide synthase (iNOS and eNOS) expression levels in the hyperthyroid heart (Oztay et al., 2007). However, the oxidized low-density lipoprotein-associated down-regulation of eNOS is declined by CoQ10 (Tsai et al., 2012). In addition, this coenzyme, dissolved in corn oil, increases the aortal eNOS activity in acrylonitrile-related vascular endothelial abnormalities in rats (Guo et al., 2011).

In another study, CoQ10 (50 mg/kg) increased the total number of spike-wave discharges (SWDs) but did not change the mean duration of SWDs. CoQ10 (100 and 200 mg/kg) increased both the total number and the mean duration of SWDs abolished by coadministration of 7-nitroimidazole (7-NI, NOS inhibitor). Coadministration of l-arginine (l-Arg, an essential substrate for the synthesis of NO) (500 and 1,000 mg/kg) enhanced the CoQ10 effect on the total number of SWDs but not on its mean duration. Therefore, the effect of CoQ10 on seizure was attenuated by the NOS inhibitor. Furthermore, based on the electrophysiological evidence, CoQ10 administration increased the absence seizures through the stimulation of the neuronal NOS (Gunes et al., 2019). Therefore, the inhibition of NOS decreased the seizure activity of CoQ10.

In contrast, subchronic oral application of CoQ10 (100 mg/kg or more) enhanced time latencies to the onset of myoclonic jerks and clonic seizures induced by intraperitoneal pentylenetetrazole (PTZ) and at the doses of 25 mg/kg or more augmented the seizure threshold provoked by intravenous injection of PTZ. Subchronic doses of CoQ10 (50 mg/kg or more) reduced the tonic seizures induced by PTZ or electroshock. This antiseizure effect of subchronic CoQ10 was attenuated by the NOS inhibitor (Sattarinezhad et al., 2014). Therefore, the interaction between NO and subchronic CoQ10 in antiseizure activity is possibly accomplished by the induction of NOS.

### The effects of CoQ10 on MS

**Figure 4:**
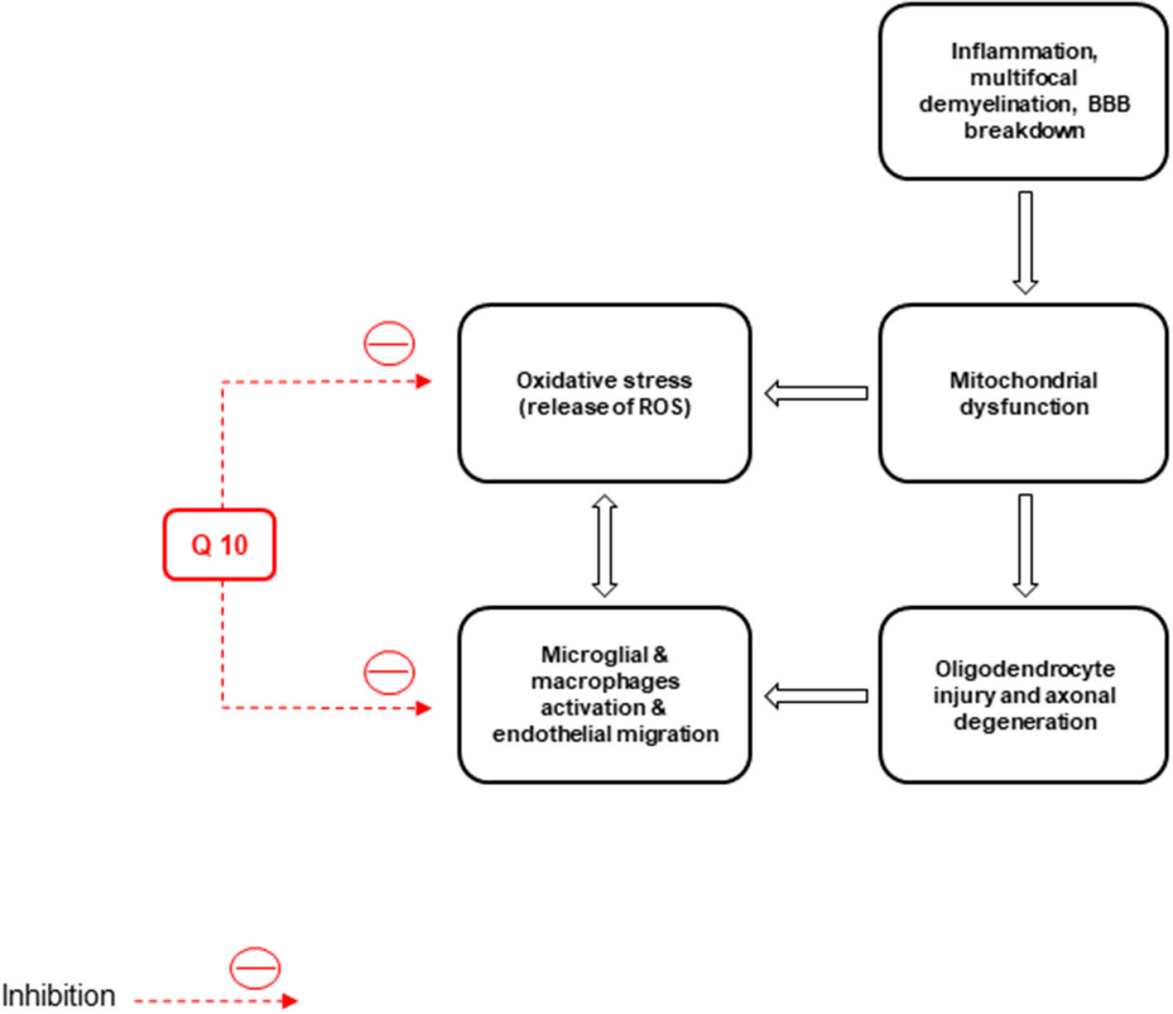
Inflammation, neuronal demyelination, mitochondrial dysfunction, destruction of axons and oligodendrocytes, and oxidative stress are the main pathological causes of multiple sclerosis (MS). CoQ10 improves the disease by reducing the activity of microglia and macrophages and the production of reactive oxygen species (ROS).

Basically, non-inflammatory mechanisms, such as mitochondrial dysfunction cause MS (Kalman et al., 2007). Free radicals play a role in MS pathogenesis and enhance the transendothelial migration of leukocytes resulting in oligodendrocyte injury and axonal degeneration (Van Horssen et al., 2011). Macrophages produce free radicals, including NO, ROS, reactive nitrogen species, microglia, and astrocytes, which all damage the neurons, axons, myelin, and oligodendrocyte (Lee et al., 2012b) (Figure 4).

Also, inflammation, multifocal demyelination, loss of oligodendrocytes, breakdown of the blood-brain barrier (BBB), neural and axonal injury, and oxidative stress are the causes of MS (Van der Walt et al., 2010; Riccio, 2011). Inflammatory markers, ROS, and matrix metalloproteinases (MMPs), as the factors to enhance BBB permeability, can be released by the infiltrated activated leukocytes in MS cases (Larochelle et al., 2011). Pro-inflammatory factors, like TNF-α, IL-1, IL-6, and interferon (IFN)-γ, increase the cerebrospinal fluid, serum, and brain lesions in MS cases. They have relatively low concentrations of transforming growth factor-β and IL-4 (Miller et al., 1998; Spooren et al., 2011).

Relapsing-remitting MS (RRMS), progressive-relapsing MS, primary progressive MS, and secondary progressive MS are different MS types (Adamczyk-Sowa et al., 2012). RRMS is linked to immune-mediated reactions, like white matter inflammation, microglial activation, and cell infiltration in the CNS (Van Horssen et al., 2011; da Silva Fernandes et al., 2012). T cells of the T-helper type 1 (Th1) macrophages play a role in the immunopathogenesis of the CD4+ demyelination, whereas remission of the disease is induced by T cells of the Th2 and Th3 phenotypes (Miller et al., 1998). The immune system plays a role in the development of depression associated with MS.

Pro-inflammatory cytokines, like TNF-α, induce weight loss, anorexia, anxiety, locomotor retardation, and reduced social exploration (Kidd, 2003). CoQ10 supplementation (60–150 mg/d) reduced inflammatory cytokines in the serum of human models (Lee et al., 2012a). Schmelzer et al. (2009) reported that the lipopolysaccharide (LPS)-related pro-inflammatory markers and chemokines decreased following preincubation of human THP-1 cells using ubiquinol-10 (QH2). Fouad and Jresat prepared CoQ10 (i.p. injections, 10 mg/kg) supplementation in a 1% aqueous solution of Tween 80. This antioxidant could reduce NF-κB and iNOS expression levels in the rats' livers (Fouad and Jresat, 2012). Treatment with CoQ10 (150 mg/kg/day) via gavage once a day for 12 weeks, could alleviate stress oxidative status caused by Cuprizone (CPZ) and significantly inhibit inflammatory biomarkers. CoQ10 enhances remyelination in the CPZ model (Khalilian et al., 2021). CoQ10 treatment (500 mg/day) improved depression and fatigue in MS patients (Sanoobar et al., 2016). Clinical symptoms in animals with experimental autoimmune encephalomyelitis (EAE) were markedly reduced (*P* < 0.05) by CoQ10 compared to controls. Also, the TNF-α level showed a significant decrease following CoQ10 administration (10 mg/kg/three weeks) vs. IL-10. The TH1/TH2 ratio in CoQ10-treated animals showed a significant decrease than in non-treated animals (*P* < 0.01) (Soleimani et al., 2014). In a randomized, double-blinded, placebo-controlled trial, patients treated with CoQ10 showed a significant elevation in SOD activity (*p* = 0.013) and a reduction in MDA concentrations (*P* = 0.003) than controls. Although CoQ10 supplementation significantly affected plasma TAC values (*p* = 0.010), no significant difference was found between both groups. CoQ10 supplementation had no effect on GPx activity (Sanoobar et al., 2013).

### The effects of CoQ10 on PD

PD is caused by the degradation of dopamine (DA) neurons in the SNpc. Over 95% of PD patients are sporadic and PD is developed in cases older than 60 years (Steece-Collier et al., 2002; Corti et al., 2005). The symptoms of this disorder include resting tremors, postural instability, rigidity, and bradykinesia (Colnat-Coulbois et al., 2005). Although the causes of sporadic have not yet been discovered, different environmental risk factors, like neurotoxins can be involved (Di Monte, 2003). PD-like symptoms in experimental animals are induced by environmental toxins, including paraquat (N, N′-dimethyl-4,4′-bipyridinium dichloride), rotenone, and maneb (Betarbet et al., 2002). Such neurotoxins inhibit complex I in the mitochondrial ETC. In normal circumstances, DA neurons also face a high level of oxidative stress because of ROS generation during DA metabolism (Dexter et al., 1994).

ATP depletion and oxidative stress production cause neuronal death. Oxidative stress, activation of the microglia, neuroinflammation, mitochondrial damage, protein aggregation due to defective clearance, and autophagic stress are the major events in the pathophysiology of PD (Kones, 2010). The disparity in mitochondrial dynamics causes augmentation of neuronal loss observed in PD patients (Srivastava, 2017). One of the main non-motor symptoms of the disease is PD with mild cognitive impairment (PDMCI). The early identification of PDMCI and treatment of this disease are of critical importance to improve the quality of life and prognosis in PD patients (Kwon et al., 2022).

The levodopa-3,4-dihydroxyphenylalanine (L-DOPA) administration is the initial treatment for PD (Nutt, 1990). Diminution of movement owing to delayed movement initiation (akinesia) is an important reason for disability in PD (Lundblad et al., 2002). L-DOPA pharmacotherapy can alleviate such symptoms. Moreover, prolonged treatments in the majority of patients lead to drug-induced abnormal involuntary movements (dyskinesia) (Cenci, 2007).

Currently, PD cannot be cured; however, neuroprotectants reduce the rate of neurodegeneration and improve the quality of life (Koller and Cersosimo, 2004). CoQ10 has shown neuroprotective activity in some neurodegenerative diseases, like PD (Mancuso et al., 2010; Wear et al., 2021). Screening for oxidative stress markers in patients with neurodegenerative disease, such as PD showed lower CoQ10 concentrations and higher lipoprotein oxidation levels in the cerebrospinal fluid, plasma, and brain cortex than in non-affected cases. Affected patients showed an increase in the levels of mitochondrial oxidative stress due to low CoQ10 levels because CoQ10 administration could improve the clinical symptoms of some patients (Jing et al., 2015). Thus, antioxidants, like CoQ10 and vitamin E, are used in both preclinical investigations and clinical trials using animal models (Shults et al., 2002; Beal, 2003; Mcdonald et al., 2005; Cleren et al., 2008; Kadian et al., 2022). In a model of PQ-related neurodegeneration in male Long-Evans rats, the water-soluble CoQ10 [WS-CoQ10; 50 mg in PBS (phosphate-buffered saline)] in drinking water was used to counteract the toxic effect of PQ. PQ induction resulted in oxidative stress and the lack of DA neurons in SNpc, which can affect the motor skill of the animals during the rotarod test. Such PD-like behavioral symptoms showed an improvement in rats treated with WS-CoQ10 added to the drinking water (Somayajulu-Nitu et al., 2009). WS-CoQ10 is not natural but can be artificially prepared. The natural CoQ10 is lipid-soluble (Parmar et al., 2015).

**Figure 5:**
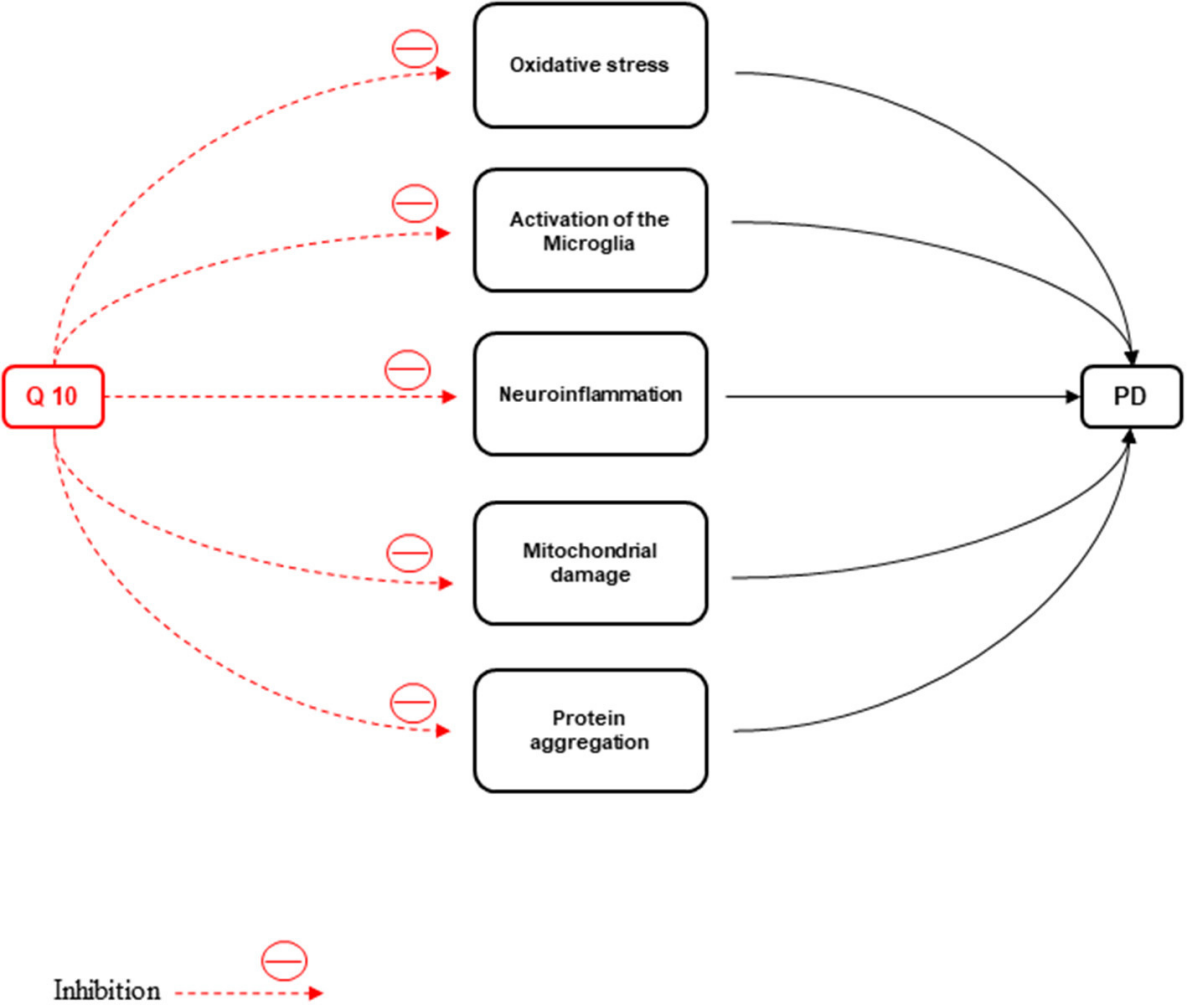
CoQ10 has a neuroprotective effect against Parkinson's disease by inhibiting inflammation, oxidative stress, activation of microglia, protein accumulation, and mitochondrial damage.

In another study, the neuroprotective effect of Ubisol Q10 (including 50 g/ml of CoQ10 and 150 g of PTS/ml) was assessed in the DJ-1/1-methyl-4-phenyl-1,2,3,6-tetrahydropyridine (MPTP) model of PD through histochemical and behavioral approaches. Application of Ubisol-Q10 could remarkably offset the neurotoxicity and ameliorate motor impairment by MPTP (Muthukumaran et al., 2014). The combination of creatine and CoQ10 treated the MPTP-associated PD model in mice, suppressed the loss of neurons containing tyrosine hydroxylase in the SNpc, and also significantly decreased LPO damage and α-synuclein accumulation in the neurons of this area but did not improve the loss of the dopaminergic neurons (Yang et al., 2009). CoQ10 (200 mg/kg/day) provided through the addition of peanut oil (glycerol could not be well-tolerated by mice during preliminary assessments) showed more effectiveness in a mouse model of PD caused by MPTP compared to oxidized CoQ10 at a similar dose. Accordingly, the elevated plasma level of CoQ10 following ingestion of the reduced form can be absorbed more effectively (Cleren et al., 2008) in comparison to the oxidized form. Treatment with CoQ10 (200 mg) for 26 weeks showed a considerable improvement in oligoasthenoteratozoospermia outcomes, which was associated with mitochondrial dysfunction in male subjects (Safarinejad et al., 2012) (Figure 5). In a clinical study, patients with PD consumed CoQ10 at three doses of 300, 600, and 1,200 mg/d for 60 days. The results showed that the first two doses were ineffective and the dose of 1,200 mg/d had healing effects (Shults et al., 2002). Furthermore, in a double-blind study, about 609 patients used CoQ10 at 400 mg daily for 6 months. CoQ10 had few toxic effects on HD, but its long-term treatment and high dose did not reduce the symptoms of the disease (McGarry et al., 2017). To gain neuroprotection, the prophylactic treatment was designed using CoQ10 (200 mg/kg (intraperitoneal) dissolved in saline) two times a week for three consecutive weeks and 30 min prior to paraquat (PQ) exposure. Furthermore, therapeutic interventions using CoQ10 in mice subjected to PQ (24 h following exposure), two times per week through 3 weeks, halted behavioral deterioration and ongoing neurodegeneration. The outcomes of the sustained treatment with CoQ10 for 3 weeks were compared to L-DOPA as the standard drug of choice. CoQ10 caused a notable improvement in most of the behavioral tests and reduced protein carbonyl content in the brain, principally when it was started before rather than after PQ induction of PD. In addition, water-soluble CoQ10 restored mitochondrial morphology and decreased fragmentation and consequently, mitochondrial fusion and improved mitochondrial dynamics, confirming the protective effect of CoQ10 against rotenone (PD-mimicking toxin) toxicity. Modulation of the fission/fusion index is therapeutically useful for the treatment of PD. Thus, water-soluble CoQ10 can be used to treat PD and is effective in other diseases due to mitochondrial dysfunction (Li et al., 2017). Therefore, CoQ10, which defends against mitochondrial damage, makes the progression of PD slow, mostly when started as prophylactic treatment (Attia and Maklad, 2018).

Intrastriatal delivery of CoQ10 at a mean rate of 1.8 and 2.6 μg daily, particularly in combination with implantable devices for deep brain stimulation or convection-enhanced delivery, can be effective to prevent neurodegeneration in PD (Park et al., 2020). Dietary CoQ_10_ supplementation (at 60 and 120 mg/kg of feed; i.p. along with oral saline) showed significant effectiveness in chlorpromazine -induced Parkinsonism-like alterations in mice (Onaolapo et al., 2021). A systematic review study showed decreased CoQ10 levels in the cerebellar cortex, platelets, and lymphocytes, increased total and oxidized CoQ10 levels in the cerebrospinal fluid, and a non-significant trend toward decreased serum/plasma CoQ10 levels in PD patients. Patients with multiple system atrophy (MSA) showed decreased CoQ10 levels in the cerebellar cortex, serum/plasma, cerebrospinal fluid, and skin fibroblasts. Patients with Lewy body dementia (LBD) showed decreased cerebellar cortex CoQ10 and those with progressive supranuclear palsy (PSP) had decreased CoQ10 levels in the cerebrospinal fluid (Jiménez-Jiménez et al., 2022).

Neurodegenerative disorders, including PD (Shults et al., 2002), amyotrophic lateral sclerosis (Ferrante et al., 2005), and HD can be treated with CoQ10 (Flint Beal and Shults, 2003). According to Yang et al. (2009), coadministration of 1% CoQ10 and the control diet, including Purina rodent chow and creatine, reduced MDA concentration (an indicator of LPO and oxidative damage) in substantia nigra pars compacta (SNpc) and showed a therapeutic effect on HD and PD (Prajapati et al., 2017).

### The effects of CoQ10 on stroke

Stroke is the third leading cause of mortality after heart disease and cancer. Three-quarters of stroke patients report an ischemic stroke, which can be due to blood vessel obstruction caused by a clot. Considerable advances have been made in neuropharmacology, but the only clinically effective treatments are acetylsalicylic acid and tissue plasminogen activator (Longa et al., 1989). Nonetheless, stroke-related mortality and morbidity rates are still high, and there is a need for the development of new treatments. Inflammation, excitotoxicity, oxidative stress, and apoptosis necrosis, are the main factors associated with lesion progression after ischemia (Ord et al., 2013). The efficacy of recanalization 3 h after limiting the onset of stroke symptoms has been approved in many patients.

**Figure 6:**
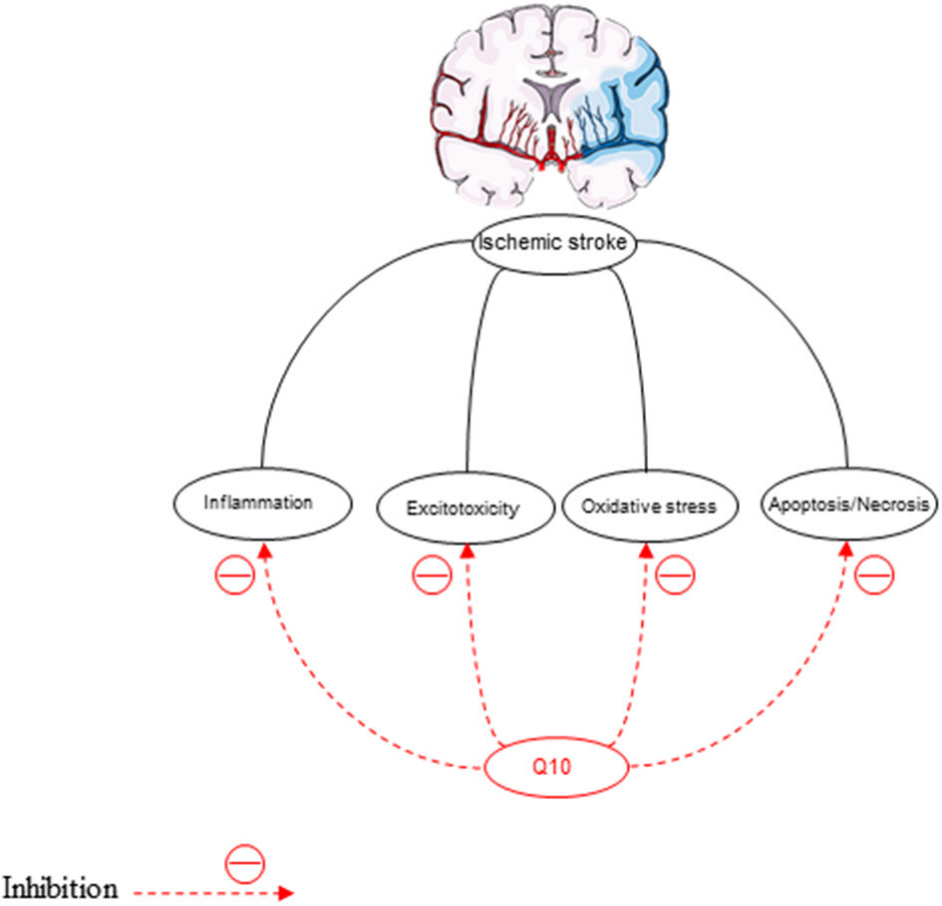
The neuroprotective effects of CoQ10 on ischemic stroke.

Oxidative stress is the pathological mechanism of cerebral ischemia (Rodrigo et al., 2013) (Figure 6). In the ischemic/reperfusion injury, excessive reactive oxygen formation is unique. Inhibition of antioxidants damages the structure and function of cells (Simani et al., 2018). Therefore, nerve cells should be protected against oxidative stress (Flint Beal and Shults, 2003; McCarthy et al., 2004). MDA is the latest product of LPO that is increased in ischemic stroke patients depending on the infarct size, the stroke severity, and the patient's outcome (Allen and Bayraktutan, 2009). Therefore, elevated concentrations of MDA in ischemic stroke patients have been reported in many studies (Simani et al., 2018). The decreased superoxide dismutase (SOD) activity in acute ischemic stroke has been observed in previous studies (Cherubini et al., 2000; Demirkaya et al., 2001; Milanlioglu et al., 2016). This antioxidant enzyme, SOD, can reduce ROS levels (Gupta et al., 2009). CoQ10 scavenges superoxide radicals for the production of oxygen and H2O2 (Lee et al., 2012a).

Different cerebral ischemia models have shown promising therapeutic effectiveness for the constant administration of CoQ10 (Obolenskaia et al., 2020). However, the treatment of such urgent conditions, such as ischemic stroke should be performed with drugs through intravenous injections. The neuroprotective effect of the solubilized CoQ10 (30 mg/kg) dissolved in saline and injected intravenously into animals with cerebral ischemia has been assessed in several studies (Belousova et al., 2016; Obolenskaia et al., 2020). The expression of genes associated with metabolism and intracellular signaling, embryogenesis, cell differentiation, and production of cholesterol and proinflammatory factors, including TNFα is affected by CoQ10 (Groneberg et al., 2005; Schmelzer et al., 2008). UbiA prenyltransferase domain-containing protein 1 (UbiAd1) is involved in CoQ10 generation and can catalyze the conversion of vitamin K1 into vitamin K2 (Mugoni et al., 2013; Povarova et al., 2018). Recently, the neuroprotective impact of vitamin K2 (menaquinone isoform) has been considered (Shearer and Newman, 2014).

Some treatments, including statin use, have been suggested for transient ischemic attack (TIA) (Gargano et al., 2011; Kernan et al., 2014). Several experimental (García-Bonilla et al., 2012) and clinical (Ní Chróinín et al., 2013) examinations have supported the effectiveness of statins in the secondary prevention of stroke outbreaks (Nasoohi et al., 2019). In other studies, the levels of blood CoQ10 in patients were reduced after the administration of atorvastatin (Rundek et al., 2004; Mabuchi et al., 2007), whereas CoQ10 levels showed no reduction in tissues (Rundek et al., 2004). However, CoQ10 in the blood may disturb BBB following an ischemic insult. Atorvastatin exerts its degenerative effects through a decrease in CoQ10 and these effects are associated with the antioxidant-oxidant defense mechanism. Furthermore, co-administration of CoQ10 (200 mg/kg/day; PO/30 days dissolved in almond oil) and atorvastatin could improve stroke outcomes (Nasoohi et al., 2019). In an interventional study, serum CoQ10 levels significantly increased in the supplement-treated acute ischemic stroke (AIS) patients compared to the placebo group. Moreover, CoQ10 (300 mg/day) supplementation significantly improved Mini-Mental State Examination (MMSE) and National Institute of Health Stroke Scale (NIHSS) scores. Nonetheless, no significant difference was found in the Modified Ranking Scale score and MDA, SOD, and glial fibrillary acidic protein (GFAP) levels between the two groups (Ramezani et al., 2020b).

### The effects of CoQ10 on LHON and SCAR9

LHON is an acute/subacute painless loss of central vision (Nikoskelainen, 1984; Novotny et al., 1986; Riordan-Eva et al., 1995). Molecular analysis has shown that primary mutations for this disease are point mutations in mitochondrial DNA (mtDNA) at positions 3,460, 11,778, and 14,484 (Wallace et al., 1988; Harding et al., 1995; Puomila et al., 2007). LHON can be associated with movement disorders, spastic paraparesis, cardiac arrhythmia, peripheral neuropathy, and skeletal abnormalities (Shoffner et al., 1995). Progressive visual loss with permanent centrocecal scotoma has been reported in most affected patients (Lessell et al., 1983; Stone et al., 1992; Tanaka et al., 1998). Some patients have symptoms, including ataxia, tremor, posterior column dysfunction, corticospinal tract dysfunction, dystonia, and extrapyramidal rigidity. LHON is associated with numerous neurologic disorders (Chariot et al., 1999).

CoQ10 is effective in the treatment of patients with mitochondrial diseases, such as chronic progressive external ophthalmoplegia (CPEO), Kearns-Sayre syndrome (KSS), and other mitochondrial encephalomyopathies (Ogasahara et al., 1986; Zierz et al., 1989; Chen et al., 1997; Sobreira et al., 1997). In a study, patients received CoQ10 orally for 4 months, and its dose increased from 90 and 160 to 200 mg/day. CoQ10 caused a rapid improvement in visual acuity in these patients (Kuo et al., 2001). In another study, treatment was initiated with 250 mg CoQ10 per day and multiple vitamins, including tocopherol (500 mg), vitamin C (150 mg), vitamin K3 (10 mg), thiamine (10 mg), and riboflavin (10 mg). Gradual improvement in movement disorders occurred within a year. The lactate/pyruvate ratio was normalized at nine, and there were no changes in visual function. Moreover, lesions of the subthalamic nuclei almost entirely disappeared (Chariot et al., 1999).

ARCA2 and SCAR9 is a kind of hereditary CoQ deficiency. This rare ataxia is due to mutations in the aarF-domain-containing kinase 3 (ADCK3) gene as an ortholog of yeast coq8 (Lagier-Tourenne et al., 2008; Mollet et al., 2008). ARCA2 is known for slow progressive gait impairment, exercise intolerance, cerebellar atrophy, epilepsy, and intellectual disability (Mignot et al., 2013). The deficiency of CoQ10 causes MRC disorder (Hargreaves, 2021). A decrease in CoQ10 concentrations in tissues or cultured cells due to biallelic mutations in each of COQ2, COQ4, COQ6, COQ7, COQ8A, COQ8B, COQ9, PDSS1, and PDSS2 genes (COQ genes) involved in the CoQ10 biosynthesis can be observed in this kind of ataxia (Emmanuele et al., 2012; Laredj et al., 2014; Quinzii et al., 2014). Patients with ADCK3 mutations experience a marked improvement following 3 weeks of oral supplementation with CoQ10 (500 mg two times a day) (Shalata et al., 2019).

## Controversial effects of CoQ10 on diseases

**Figure 7:**
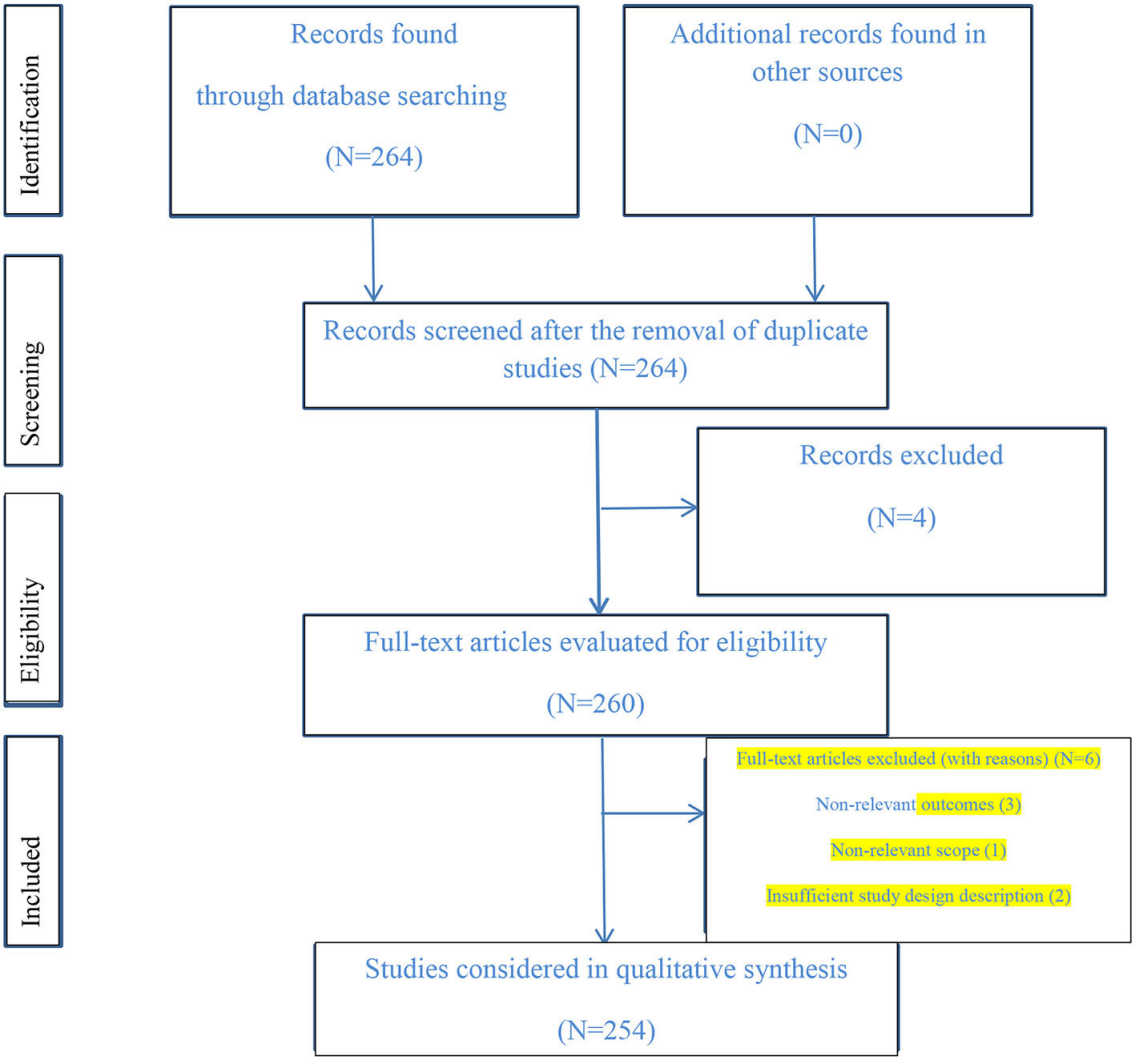
The PRISMA flow diagram of the screening and selection of the study.

The purpose of this article was to review the studies using experimental and clinical treatments (Figure 7). Experimental treatments have often been studied in animal models with remarkable results. In addition, clinical studies using a specific dose and duration of treatment have been effective. However, CoQ10 has no effect on some of the symptoms of the disease. The antidepressant effect of this neuroprotective agent has not yet been studied in patients with MS (Sanoobar et al., 2016). Also, in an animal study, contrary to the antioxidant effects of CoQ10, it led to an increase in the aortal eNOS activity in vascular endothelial abnormalities caused by acrylonitrile in rats (Guo et al., 2011). The neuroprotective properties of CoQ10 in the intrahippocampal kainate model of TLE have not yet been identified and more research is needed (Baluchnejadmojarad and Roghani, 2013). In a clinical study, patients with PD were treated with CoQ10 at three doses of 300, 600, and 1,200 mg/d, and only the dose of 1,200 showed a healing effect (Shults et al., 2002). In addition, long-term, high-dose CoQ10 therapy did improve the symptoms of HD (McGarry et al., 2017). In an intervention study on AIS patients, there were no statistically significant differences in the Modified Ranking Scale score, and MDA, SOD, and GFAP levels between the two placebo and supplement-treated groups (Ramezani et al., 2020b). A recent study on patients with LHON showed no changes in visual function after the administration of CoQ10 (250 mg) and other vitamins in the bilateral pallor of the optic disks (Chariot et al., 1999). According to the mentioned studies and their results, more relevant studies are needed in the future.

## Conclusions

CoQ10 as an antioxidant and neuroprotective agent can play a role in the treatment of neurological disorders. Although neurological diseases cannot be treated effectively, CoQ10 deficiency is involved in the pathogenesis of epilepsy, stroke, MS, depression, PD, AD, LHON, ARCA2, and SCAR9. More clinical and experimental studies are needed using electrophysiological and behavioral evaluation, genetic targeting, and molecular imaging.

## Author contributions

SB, RH, SS, MK-A, AM, MR, and AK literature review and drafting the manuscript. SB and AK critically revision of the manuscript. All authors read and approved of the final manuscript.

## Glossary

AD, Alzheimer's Diseases; ARCA2, Autosomal Recessive Cerebellar Ataxia 2; ATP, Adenosine triphosphate; Aβ, β-amyloid; AChE, Acetylcholinesterase; ADCK3, AarF-domain-containing kinase 3; BD, Bipolar disorder; BBB, Blood-brain barrie; βNF, β-naphthoflavone; CYP, Cytochrome P450; CoQ10, Coenzyme Q10; CPEO, Ophthalmoplegia; CUMS, Chronic unpredictable mid stress; CPZ, Cuprizone; DA, Dopamine; DMSO, Dimethyl sulfoxide; Drp1, Dynamin-related protein 1; ETC, Electron transport chain; EtOH, Ethanol; EAE, Experimental autoimmune encephalomyelitis; eNOS, Endothelial nitric oxide synthase; FCCP, Carbonyl cyanide p-trifluoro-methoxyphenyl; Fis1, Fission protein 1; FM, Fibromyalgia; FST, Forced swim test; GFAP, Glial fibrillary acidic protein; GPx, Glutathione peroxidase; H2O2, Hydrogen peroxide; HD, Huntington disease; HIP, Hippocampus; HMG-CoA, Hydroxymethylglutaryl coenzyme A; HPLC, high-performance liquid chromatography; IDO-1, Indoleamine 2, 3-dioxygenase 1; IL-1β, Interleukin-1β; (IFN)-γ, Interferon; IMM, Inner mitochondrial membrane; iNOS, Induced nitric oxide synthase; ICV, Intracerebroventricular; KSS, Kearns-Sayre syndrome; LHON, Leber's hereditary optic neuropathy; LPO, Lipid peroxidation; LTP, Long-term potentiation; LPS, Lipopolysaccharide; L-DOPA, Levodopa-3,4-dihydroxyphenylalanine; LBD, Lewy body dementia; MDA, Malondialdehyde; MMPs, Matrix metalloproteinases; MPTP, 1-methyl-4-phenyl-1,2,3,6-tetrahydropyridine; MSA, Multiple system atrophy; MRC, Mitochondrial respiratory chain; MtDNA, Mitochondrial DNA; MS, Multiple sclerosis; NO, Nitric oxide; ONH, Optic nerve head; PD, Parkinson's Diseases; PSP, Progressive supranuclear palsy; PS-1, Presenilin 1; PTZ, Pentylenetetrazole; PDMCI, PD mild cognitive impairment; PQ, Paraquat; PH&M, Physical and mental activities (PH&M); PFC, Prefrontal cortex; QH2, Ubiquinol-10; ROS, Reactive oxygen species; RET, Rearranged during transfection; RRMS, Relapsing-remitting MS; SCAR9, Spinocerebellar ataxia autosomal recessive 9; SE, Status epilepticus; SNpc, Substantia nigra pars compacta; SOD, Superoxide dismutase; STZ, Streptozotocin; SWDs, Spike-wave discharges; TBARS, Thiobarbituric acid reactive substances; TNF-α, Tumor necrosis factor-alpha; TLE, Temporal lobe epilepsy; Th1, T-helper type 1; TIA, Transient ischemic attack; TAC, Total antioxidant capacity; UCPs, Uncoupling proteins; UQ, Oxidized ubiquinone; UbiAd1, UbiA prenyltransferase domain-containing protein 1; UQH2, Reduced ubiquinol; VIN, Vinpocetin; WS-CoQ10, Water-Soluble CoQ10; l-Arg, l-arginine; 7-NI, 7- nitroimidazole.
